# Potential Application of Marine Fucosyl-Polysaccharides in Regulating Blood Glucose and Hyperglycemic Complications

**DOI:** 10.3390/foods12132600

**Published:** 2023-07-05

**Authors:** Luying Tang, Mengshi Xiao, Shenyuan Cai, Haijin Mou, Dongyu Li

**Affiliations:** College of Food Science and Engineering, Ocean University of China, No. 1299 Sansha Road, Qingdao 266003, China; tangluying@stu.ouc.edu.cn (L.T.); 18227591863@163.com (M.X.); 18986507217@163.com (S.C.); mousun@ouc.edu.cn (H.M.)

**Keywords:** marine fucosyl-polysaccharides, bioactivity, blood glucose, structure–activity relationship, hyperglycemic complications

## Abstract

Diabetes mellitus (DM) has become the world’s third major disease after tumors and cardiovascular disease. With the exploitation of marine biological resources, the efficacy of using polysaccharides isolated from marine organisms in blood glucose regulation has received widespread attention. Some marine polysaccharides can reduce blood glucose by inhibiting digestive enzyme activity, eliminating insulin resistance, and regulating gut microbiota. These polysaccharides are mainly fucose-containing sulphated polysaccharides from algae and sea cucumbers. It follows that the hypoglycemic activity of marine fucosyl-polysaccharides is closely related to their structure, such as their sulfate group, monosaccharide composition, molecular weight and glycosidic bond type. However, the structure of marine fucosyl-polysaccharides and the mechanism of their hypoglycemic activity are not yet clear. Therefore, this review comprehensively covers the effects of marine fucosyl-polysaccharides sources, mechanisms and the structure–activity relationship on hypoglycemic activity. Moreover, the potential regulatory effects of fucosyl-polysaccharides on vascular complications caused by hyperglycemia are also summarized in this review. This review provides rationales for the activity study of marine fucosyl-polysaccharides and new insights into the high-value utilization of marine biological resources.

## 1. Introduction

**Figure 1:**
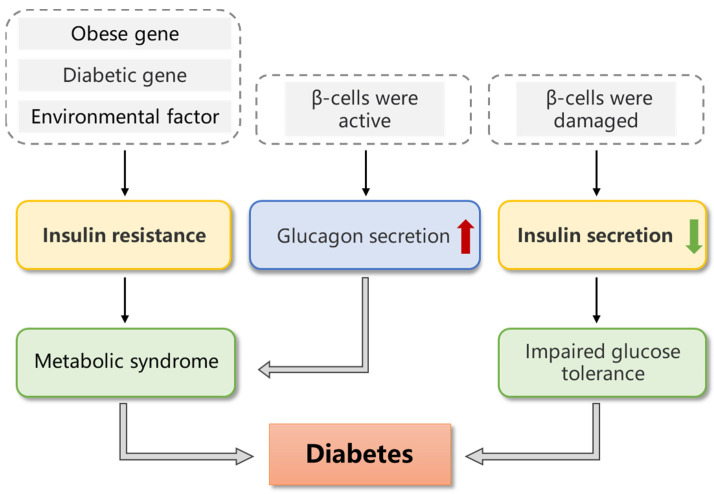
Pathogenesis of diabetes mellitus.

The blood glucose level refers to the venous plasma glucose level. A normal blood glucose value refers to the blood glucose range of 3.9–6.1 mmol/L in fasting conditions. According to the criteria, a blood glucose level <3.9 mmol/L is hypoglycemia, and blood glucose >10 mmol/L is hyperglycemia [1,2]. A high blood glucose level induced by eating many carbohydrates and stressful states, such as having a cold, pneumonia, acute heart attack or severe illness, is known as physiological hyperglycemia [3]. Insufficient or/and low-level insulin secretion can disrupt carbohydrate and lipid metabolism in the body, finally leading to diabetes mellitus (DM). DM is a chronic disease mainly characterized by hyperglycemia [4,5]. DM can be divided into four categories according to the different causes: type I diabetes mellitus (T1DM), type II diabetes mellitus (T2DM), gestational diabetes mellitus (GDM) and other special types of diabetes mellitus (Table 1/Figure 1). Among them, T2DM is the most common type, accounting for about 90% of the incidences [6,7,8,9,10,11,12,13,14].

Although the causes and characteristics of DMs differ, they all involve glucose and insulin. Glucose is mainly derived from food and the breakdown of hepatic glycogen [15]. Insulin is a protein hormone secreted by β-cells, which can promote glucose to enter the cells and glycogen synthesis [16]. Insulin resistance occurs when β-cells cannot produce enough insulin due to impaired insulin sensitivity [17]. Long-term hyperglycemia can also induce chronic complications, including coronary heart disease, arteriosclerosis, renal failure, retinopathy, neuropathy, osteoporosis and gastrointestinal disorders [18]. Most of these complications are associated with vascular lesions: (1) hyperglycemia can cause vascular lesions, (2) impairing insulin-mediated glucose metabolism and insulin secretion, which contribute to T2DM [19]. Complications would lead to increased morbidity and mortality. In recent years, the number of DM patients has continued to increase, and they are younger. According to the World Health Organization (WHO), diabetes has become the third most common disease in the world after tumors and cardiovascular diseases. Epidemiologists predict that by 2045, the number of DM patients will rise to 783 million worldwide [20]. Its high prevalence has a profound impact on socio-economic development [21]. Therefore, it is essential to conduct in-depth research on blood glucose control and diabetes prevention.

Currently, the oral drugs for T2DM mainly include biguanides, thiazolidinediones and sulfonylureas [22,23]. Among them, metformin alleviates blood glucose by inhibiting the output of hepatic glycogen and increasing the sensitivity of peripheral tissues to insulin [24]; glimepiride can promote insulin secretion and convert blood glucose into glycogen [25]. These exhibited good hypoglycemic effects when used alone or in combination with insulin, but there are still some problems, such as they may not play the expected role and may have side effects on a patient’s health [25,26]. As a result, researchers are increasingly looking to find alternative sources of natural resource-based glycemic drugs to delay hyperglycemic complications, mitigate side effects and reduce costs [4,7]. In recent years, with the development and utilization of marine biological resources, some active ingredients isolated from marine organisms, such as marine polysaccharides, have been proved to have the effects of lowering blood pressure/glucose/lipid, anticoagulant and immune regulation, thus attracting widespread attention [27,28,29,30,31]. Marine fucosyl-polysaccharides extracted from algae and sea cucumbers are a kind of fucose-containing sulphated polysaccharide. Recent studies have shown that these polysaccharides have significant hypoglycemic effects both in vivo and in vitro; however, the structure of marine fucosyl-polysaccharides and the mechanism of their hypoglycemic activity are not yet clear. Therefore, in this review, we summarize the marine fucosyl-polysaccharides with a hypoglycemic effect, analyze the molecular mechanism of their efficacy and further explore the relationship between the structure and activity of marine fucosyl-polysaccharides to provide an essential basis for the development and high-value utilization of marine biological resources.

## 2. Marine Polysaccharides with Hypoglycemic Activity

Natural polysaccharides, such as marine polysaccharides, have sparked a growing interest in regulating blood glucose and hyperglycemic complications [32,33,34,35]. Polysaccharides and their modified derivatives, some with medicinal capabilities, have been observed to portray non-existent toxicity and beneficial effects [36]. As natural components, marine polysaccharides have good biocompatibility and bioavailability [37]. As shown in Table 2, marine polysaccharides with good hypoglycemic effects are mainly derived from brown algae and sea cucumbers, and a few polysaccharides from red algae, green algae and microalgae sources have similar effects.

**Figure 2:**
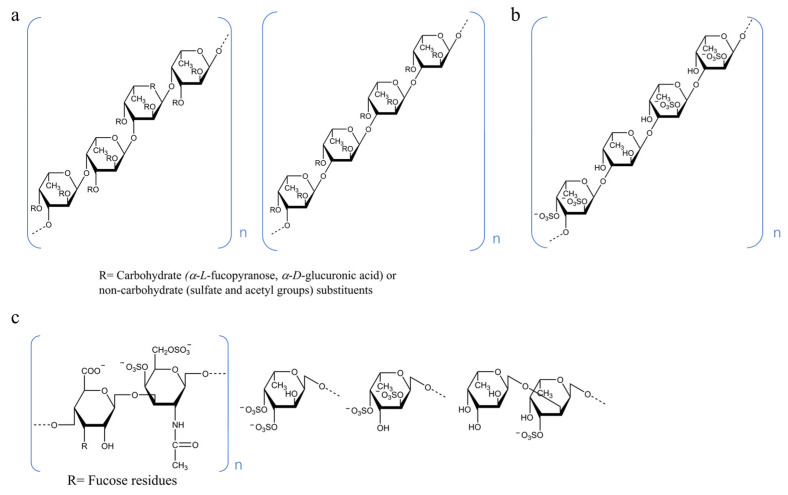
Structure of marine fucosyl-polysaccharides from brown algae and sea cucumber. (**a**) F Fucoidan (FU) from brown algae. (**b**) Sulfated fucan (SC-FUC) and (**c**) fucosylated chondroitin sulfate (SC-FCS) from sea cucumbers.

Currently, studies on the hypoglycemic activity of brown algae polysaccharides have mainly focused on the species genera, *Sargassum pallidum*, *Sargassum fusiform* and *Undaria pinnatifida*, and fucoidan (FU) extracted from them is on of marine fucosyl-polysaccharides. The structure of FU is shown in Figure 2a. There are two types of FU structures: type I chains and type II chains. The type I chain contains repeated (1→3)-α-l-fucose with sulfate groups in C2- and C4-positions, whereas the type II chain contains alternating (1→3) and (1→4)-α-l-fucose with sulfate groups in C2-, C3- and C4 positions [86]. Cao et al. showed that both the polysaccharide fractions SPP-1 and PSP-1 from *S. pallidum* exhibited significant hypoglycemic activity in vitro and could effectively inhibit α-amylase and α-glucosidase activities [44,47]; similar results were proved in other studies [45,46]. SFP-2, SFF and SFP-7-40 obtained from *S. fusiform* effectively alleviate DM through different mechanisms in vivo [51,52,57,59,62]. The fractions, LMWF, UP4 and UPP, extracted from *U. pinnatifida* have significant hypoglycemic effects in vivo [38,56,60]. Additionally, polysaccharides extracted from brown algae, such as *Ecklonia maxima*, *Macrocystis pyrifera*, *Dictyopteris divaricata* and *Laminaria japonica,* have also been shown to have hypoglycemic activity [58,63,66,87]. Polysaccharides derived from red algae have also been proven to inhibit α-amylase and α-glucosidase activities, regulate glucolipid metabolism, repair pancreatic β-cells, protect liver and kidney function and promote endogenous antioxidant enzymes in vivo [41,42,43]. ULP obtained from green alga, *Ulva lactuca,* had significant hypoglycemic effects on diabetic mice [64]. PSP3 extracted by Liu from the microalga, *Spirulina platensis,* has been shown to have prominent hypoglycemic activity in vitro and in vivo [67]. Marine fucosyl-polysaccharides from sea cucumbers are usually classified into two groups: sulfated fucan (SC-FUC) and fucosylated chondroitin sulfate (SC-FCS). As shown in Figure 2b,c, SC-FUC is made up of repetitive tetrasaccharide units formed by α (1→3) units and with a regular sulfation pattern at positions 2 and 4, and SC-FCS has a chondroitin sulfate-like backbone with branches of α-fucose linked to position 3 of β-glucuronic acid of the central core [88,89]. Marine fucosyl-polysaccharides extracted from sea cucumber species, such as *Acaudina molpadioides*, *Cucumaria frondose*, *Isostichopus badionotus*, *Thelenota ananas*, *Pearsonothuria graeffei*, *Stichopus japonicus*, *Holothuria tubulosa* and *Apostichopus japonicus,* showed hypoglycemic activity. In addition, neutral polysaccharides extracted from *Holothuria leucospilota* and *Stichopus japonicus* also have hypoglycemic effects [81,84]. In recent years, the application of low-edible-value sea cucumber for the treatment of hypoglycemia and the improvement of diabetes has received widespread attention. Zhu et al. found that marine fucosyl-polysaccharides (TAPF and CFPF) extracted from low-edible-value sea cucumber could exert hypoglycemic functions by lowering the fasting blood glucose levels, improving glucose tolerance, promoting insulin secretion, improving insulin resistance and promoting hepatic glycogen accumulation [82].

The structure and effect of marine polysaccharides from various sources are different, and most studies focus on fucose-containing sulphated polysaccharides such as fucoidan (FU), SC-FUC and SC-FCS.

## 3. Hypoglycemic Mechanisms of Marine Fucosyl-Polysaccharides

**Figure 3:**
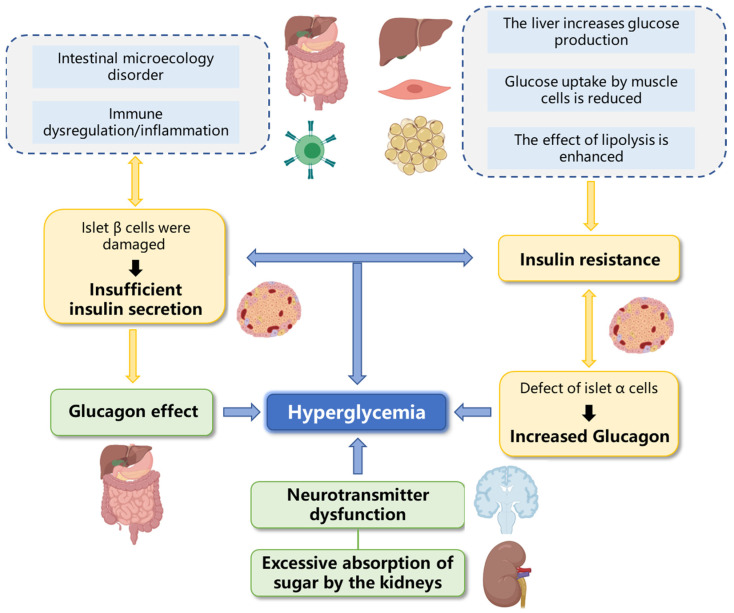
Diagram of blood glucose elevation pathways/mechanisms.

As shown in Figure 3, pathological blood glucose elevation is mainly caused by insufficient insulin secretion, insulin resistance, insufficient glucagon secretion and other factors. The hypoglycemic effect of marine fucosyl-polysaccharides is related to complex multi-pathway, multi-link and multi-target biological processes. Recent studies have shown that the mechanisms of action of marine fucosyl-polysaccharides mainly include the inhibition of digestive enzyme activity, the improvement of insulin resistance, the protection of β-cells and the regulation of intestinal microflora.

### 3.1. Inhibit Digestive Enzyme Activity

Currently, the inhibition of α-amylase and α-glucosidase activity is considered to be an effective treatment for type 2 diabetes, as well as the most direct way to lower the blood glucose level evaluated in vitro. Dietary starch consumed by the body is broken down in large amounts by turning α-amylase into oligosaccharides, such as maltose. α-glucosidase located on the brush border surface membrane of intestinal cells degrades oligosaccharides into monosaccharides absorbed by the intestinal epithelial cells, resulting in elevated blood glucose levels. The production of glucose is the cause of elevated postprandial blood glucose levels, which can cause reduced insulin sensitivity in diabetic patients, leading to severe complications and the aggravation of disease [41,90].

Many studies have shown that marine fucosyl-polysaccharides can effectively control postprandial blood glucose levels by inhibiting α-amylase and α-glucosidase activities, thus playing a role in hypoglycemia. However, due to the different time of action of the target enzyme, polysaccharides from different sources have different effects on α-amylase and α-glucosidase. PSP-1 extracted from *S. pallidum* showed a certain inhibitory activity against α-amylase and α-glucosidase in a dose-dependent manner [47]. Heng et al. synthesized selenated *S. pallidum* polysaccharide SPP derivatives (SE-SPP) with IC_50_ values of 1.579 and 0.896 mg/mL for the inhibition of α-glucosidase activity via SPP and se-SPP, respectively [48]. The IC_50_ values of the low-molecular-weight FU fraction LMW As-H obtained via enzymatic digestion were 1150 ± 10 μg/mL and 560 ± 10 μg/mL for α-amylase and α-glucosidase, respectively. For α-amylase, LMW As-H induces secondary structure changes and spatial conformational transitions in the enzyme, altering the formation of the catalytic site or preventing substrate binding, thereby affecting its activity. In contrast, LMW As-H binding only alters the microenvironment around α-glucosidase, leading to a shift in its spatial conformation, which prevents substrate binding or the formation of enzyme–substrate–inhibitor complexes, resulting in a decrease in enzyme activity [65]. In addition, some studies have shown that the inhibitory effect of FU itself on enzymes is related to its glucuronic acid structure, and free carboxyl groups can be targeted to select α-glucosidase [48]. It is worth mentioning that studies have shown that excess α-amylase causes symptoms such as bloating, flatulence and diarrhea in the body; so, polysaccharides with mild inhibitory activity against α-amylase, but a more substantial inhibitory effect on α-glucosidase, are the most desirable enzyme inhibitors [91].

### 3.2. Improve Insulin Resistance

Insulin resistance (IR) occurs when normal circulating concentrations of hormones fail to regulate in vivo glucose homeostasis in target tissues, and it occurs preferentially in the liver [16]. When the body develops IR, the insulin recognition receptor level is abnormal, decreasing the efficiency of glucose uptake and utilization. Then, the body compensates by secreting too much insulin to produce hyperinsulinemia, which leads to metabolic syndrome and T2DM [17].

**Figure 4:**
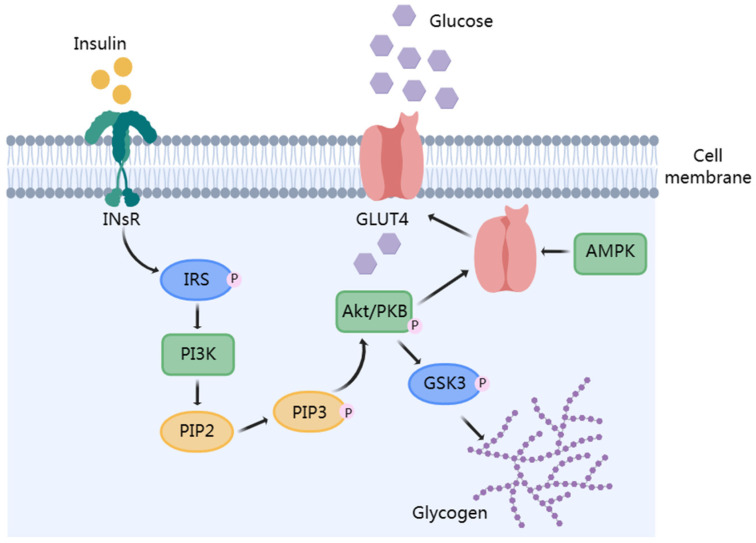
Diagram of insulin hypoglycemia through PI3K-Akt-GLUT4 pathway.

Insulin activates the PI3K-Akt signaling pathway and further regulates glycogen synthase kinase 3 (GSK3), which increases glycogen synthesis and contributes to a lower blood glucose level [16,92]. In addition, PI3K-Akt can induce glucose transporter 4 (GLUT4) expression, and the phosphorylation of PKB promotes GLUT4 expression and translocation to the cell membrane, thereby facilitating glucose transport [93]. Figure 4 shows that the two pathways can be connected. Studies have shown that marine fucosyl-polysaccharides can improve insulin sensitivity by regulating the expression of receptors in the IRS/PI3K/Akt signaling pathway, thereby improving insulin resistance. *U. pinnatifida*-derived UPPs can alleviate IR by enhancing the IRS/PI3K/Akt signaling pathway, increasing blood glucose absorption and utilization in diabetic rats and reducing endogenous glucose production [60]. *I. badionotus* SC-FUC regulates hepatic glycogen synthesis and glucose metabolism by activating the hepatic PI3K/Akt/GSK-3β signaling pathway [82]. SC-FCS extracted from *C. frondosa* activates the PI3K/Akt insulin signaling cascade, leading to total GLUT4 translocation, increasing hepatic glycogen synthesis, thereby ameliorating hyperglycemia-induced IR [72,94]. Although many studies have shown that marine fucosyl-polysaccharides can improve IR by regulating PI3K-Akt signaling pathway and related gene, the targets are not clear.

In addition, endoplasmic reticulum stress (ER) induces the inactivation of the AMP-activated protein kinase (AMPK) pathway, resulting in IR [38]. AMPK is essential in GLUT4 transport in skeletal muscle and adipocytes; the activated phosphorylation of AMPK (p-AMPK) controls blood glucose stability by inhibiting hepatic gluconeogenesis [95,96]. Jeong et al. showed that in db/db mice, LMWF acutely activated the LKB1/AMPK pathway, thereby stimulating glucose uptake and fatty acid oxidation in myocytes to improve IR [48]. Similarly, the treatment of HFSD-induced mice with SC-FCS extracted from *Cusumaria frondose* can alleviated hepatic ER stress to increase liver insulin sensitivity and improve IR [74].

### 3.3. Improve β-Cells Structure and Function

Insulin is a protein hormone secreted by the β-cells of pancreatic islets, which also promotes glycogen, fat and protein synthesis [97]. Marine fucosyl-polysaccharides can improve β-cells by increasing cell numbers, reducing oxidative stress and inhibiting apoptosis, and thus, it can improve the rate of insulin secretion. It has been shown that the relative β-cell mass is decreased by 45% and 70% in lean and obese populations with T2DM, respectively, and that β-cell apoptosis is elevated in both. The underlying mechanisms of β-cell apoptosis are currently complex and controversial, but increasing the number of β-cells is an important way to treat DM [4,98].

Various marine fucosyl-polysaccharides are effective in increasing the number of beta-cells, and thus, have a hypoglycemic or improving DM effect. Setyaningsih et al. extracted EPs from *Porphyridium cruentum* to effectively increase the number of β-cells [55]. Streptozotocin (STZ) is a specific compound that penetrates the pancreatic wall through the protein channel glucose transporter 2 (GLUT2), damages β-cells and causes autoimmunity [99]. EP protects β-cells by blocking the action of STZ on GLUT2, a glucose receptor on the β-cell membrane, via binding to GLUT2 [55]. Furthermore, SC-FCS extracted from *C. frondosa* can activate BCL-2 and Bcl-xL, leading to the inactivation of the intrinsic mitochondrial pathway, thereby reversing HFSD-induced β-cell apoptosis [73]. The homeostasis model assessment-β (HOMA-β) is an indicator of the normal function of β-cell in insulin secretion; an increase in the HOMA-β value means the reduction of pancreatic β-cell damage and the promotion of insulin secretion [96,100]. Zhu et al. found that *C. frondosa*-derived CFPF increased the HOMA-β values in T2DM rats, suggesting that CFPF effectively improved impaired β-cell functions [82]. CDDP obtained from *D. divaricata* also partially restored β-cell loss by increasing the HOMA-β levels, while improving fasting glucose, oral glucose tolerance (OGTT) and serum insulin levels in T1DM mice [63].These results suggest that marine fucosyl-polysaccharides help increase the β-cell mass and reduce β-cell dysfunction.

### 3.4. Regulate Intestinal Microflora

In recent years, research on gut microbiota has received a lot of attention. And there is evidence of a strong link between intestinal microflora and diabetes [101,102]. Altered intestinal microflora in patients with T2DM compared to those of the healthy population are characterized by a decrease in the ratio of *Bacteroidetes*/*Firmicutes* ratio and some functional bacteria (e.g., *Bifidobacterium*), accompanied by an increase in pathogenic bacteria and some endotoxin-producing Gram-negative bacteria [103]. Changes in the intestinal microflora are a characteristic feature of T2DM. The increase in the amount of pathogenic bacteria and the decrease in the diversity of the intestinal microflora weaken the function of the intestinal mucosal barrier and produce many metabolites that activate metabolic pathways and promote the development of DM [102].

Some marine fucosyl-polysaccharides can participate in the metabolism of intestinal microflora, and the metabolites produced will affect the type and quantity of intestinal microflora, and then affect glucose metabolism to regulate blood glucose [104]. Chen et al. extracted ULP-1 (100 mg/kg) from *U. lactuca* to significantly increase the abundance of intestinal microflora. It was further verified that *Lactobacillus*, *Weissella*, *Romboutsia*, *Dubosiella* and *Turicibacter* dominate the microenvironment of the intestinal microbiota administered via ULP and contribute to ameliorate the harmful effects of diabetes on the organism [64]. SFP-2 extracted from *S. fusiform* can promote the growth of *Muribaculaceae_norank*, *Akkermansia*, *Bifidobacterium* and *Lactobacillus* in the guts of diabetic rats and increase the abundance of intestinal microflora [51]. Other researchers have found similar results with FU extracted from different *Sargassum* species because the higher ratio of fucose and galactose in them favors colon microbiota fermentation, changes the abundance of probiotics and reduces the pathogenic bacteria and promotes the intestinal microflora of diabetic rats at the normal level [52,59]. The evidence suggests that short-chain fatty acids (SCFAs) act as an active signaling molecule to improve glucose homeostasis and insulin sensitivity, promoting the secretion of metabolism-related hormones from epithelial cells and participating in glucose metabolism [104]. For example, sea cucumber-derived Am-FUC significantly improved the ratio of *Bacteroidetes*/*Firmicutes* in the intestines of HFD mice and promoted the secretion of SCFAs, effectively reducing HFD-induced insulin resistance [70]. Zhao et al. showed that *H. leucospilota* polysaccharide HLP could have a mitigating effect on T2DM by increasing the production of SCFAs, such as acetic acid, propionic acid and valeric acid in mice, as well as positively regulating the hosts’ intestinal microflora [77]. In addition, marine fucosyl-polysaccharides can improve the intestinal barrier. The intestinal barrier functions to directly prevents intercellular junctions through which bacterial products pass, and it also keeps the bacteria themselves at a safe distance from the epithelial cells and helps maintain a stable microbiota composition [103]. CDDP extracted from *D. divaricata* can maintain the intestinal structure and barrier permeability by increasing the levels of insulin receptor substrate-1 (IRS-1), mucin-2 (MUC-2) and tight junction proteins (TJs), improving intestinal morphology and protecting the intestine from harmful chemicals or pathogens [63].

Research on gut microbiota in the pathogenesis of diabetes is still in its early stages. Still, it provides new insights and reliable information for treating diabetes and potential intervention strategies, and there is great potential to improve metabolic diseases, including diabetes, via intestinal microflora.

## 4. Structure–Activity Relationship (SAR)

Polysaccharide is a kind of macromolecule with a complex structure. Its biological activity is related to its monosaccharide composition, molecular weight, conformation and glycoside bond type. Nevertheless, most current studies have focused more on the structure or activity of marine fucosyl-polysaccharides and less on their SAR. The authors of the following comprehensive and extensive studies analyzed the effects of the molecular weight, sulfate groups, monosaccharide composition, conformation and glycosidic bond type of marine fucosyl-polysaccharides on their hypoglycemic activity, which facilitate the development and application of hypoglycemic drugs.

### 4.1. Molecular Weight

Molecular weight is one of the critical factors affecting marine fucosyl-polysaccharides. Among the FU, the molecular weight of FU varies from several kDa to several thousand kDa, and low-molecular-weight FU extracted from the same species is usually considered to have a better hypoglycemic effect. For example, in diabetic mice, fucoidan with molecular weights below 5 kDa have more effective hypoglycemic activity than fucoidan with 5–30 kDa does [105]. Three fractions, Up-3 (84.8 kDa), Up-4 (41.4 kDa) and Up-5 (330.7 kDa), extracted from *U. pinnatifida* have different hypoglycemic effects. At a 100 μg/mL concentration, the α-glucosidase and α-amylase inhibitory activity levels of low-molecular-weight Up-4 were higher than those of acarbose and the other two fractions [56]. FU from *Fucus vesiculosus* (2351 kDa) has no effect on α-amylase activity, but FU from *Ascophyllum nodosum* (637 kDa) has an inhibitory effect [106]. Zhan et al. found that the size of the inhibitor molecules influences the effectiveness of polysaccharide inhibitors interacting with enzymes. High-molecular-weight polysaccharides are not conducive to interactions with enzymes due to their larger spatial conformation, and the appropriate reduction of molecular weight can increase the exposure rate of the active groups in polysaccharides, thus promoting interactions with enzymes [65]. Therefore, the hypoglycemic activity of marine fucosyl-polysaccharide can be improved after a certain degree of degradation. The significant hypoglycemic benefits of many *Sargassum*-derived FUs have been analyzed before, and degraded *Sargassum* oligosaccharides (SCO) significantly reduced fasting blood glucose levels in HFSD mice and improved hepatic IR by modulating IRS1/PI3K signaling pathways [49]. The same results were found in a study on SC-FUC. Hu et al. found that degraded Am-FUC was more effective at relieving insulin resistance [85]. In recent years, marine oligosaccharides or low-molecular-weight polysaccharides have been shown to have hypoglycemic effects, the advantages of which are expressed in the stimulation of insulin secretion [107].

### 4.2. Sulfate Group

Sulfated polysaccharides contain sulfate groups on the hydroxyl groups of sugar units, including naturally extracted polysaccharides and synthetic acid derivatives of natural neutral polysaccharides [108]. Numerous scientific studies have shown that polysaccharides with a sulfate group have better biological properties than those that are not sulfated do [89,109,110,111]. Many marine fucosyl-polysaccharides contain sulfuric groups and do not need to be modified by chemical methods [112]. The content, location and pattern of sulfate groups in marine fucosyl-polysaccharides are closely related to their biological activity [113].

#### 4.2.1. Content of Sulfate Group

There is increasing evidence that sulfated polysaccharides bind more strongly to cationic proteins and are generally more bioactive due to the sulfate groups [113,114]. Sulfate groups can modify the chemical properties of polysaccharides to some extent in four main ways: (1) sulfate groups have a negative charge over a wide pH range (4–12) and can easily bind to positively charged biomolecules; (2) sulfate groups can coordinate water molecules to increase and maintain tissue hydration levels; (3) multiple sulfate groups on a single polysaccharide can promote the stretching of the solvation conformation, thus minimizing electrostatic repulsion between negative charges; (4) sulfated polysaccharides are negatively charged polymers and do not change the pH [115].

In FU, its sulfate content mainly had a range of 7.66–38.3%. The FU of *U. pinnatifida* origin completely lost its inhibitory effect on α-amylase activity after desulfurization. The sulfate content of fucoidan was increased by a chemical persulfate treatment, thus enhancing its inhibitory effect on α-amylase [116]. SPP was sulfated, and its sulfate group content was increased from 3.31% to 13.36%. Compared with the natural polysaccharide, the sulfated modified polysaccharide S-SPP1-8 increased the inhibitory activity of α-glucosidase to 98.4% (1 mg/mL) and enhanced the consumption of glucose by HepG2 cells [46]. Similarly, after removing the sulfate group of FU, the sulfate group content was less than 2.7%, and the ability to inhibit α-amylase was lost entirely [39]. Koh et al. suggested that FU inhibits α-amylase and α-glucosidase activities through electrostatic interactions with the sulfate group of FU bound to the secondary site of the enzyme–substrate complex and by increasing the viscosity of the reaction medium [117]. The above results indicate that the negatively charged sulfate group in FU can change the conformation of the digestive enzyme by binding to the positively charged amino acid in the digestive enzyme through electrostatic interaction, thus changing the catalytic ability of the digestive enzyme. Moreover, the content of the sulfate group affects its ability to inhibit digestive enzymes. However, the exact site of this electrostatic interaction and the mechanism by which the sulfate group in the sulfate polysaccharide inhibits enzyme activity are not known. On the other hand, the high viscosity of FU affects its diffusion in the solvent and increases the time required for FU to reach the enzyme. In contrast, increasing the sulfate group can reduce the polysaccharide viscosity and improve the polysaccharide solubility, thus improving the interaction effect of FU with the enzyme [68]. However, the sulfate group of the degraded galactomycin polysaccharide increased from 19.4% to 30.3%, but its ability to inhibit α-amylase was lost [39]. This suggests that the number of sulfate groups is not the only factor affecting the hypoglycemic effect of FU.

In addition, the sulfate group in marine fucosyl-polysaccharides has an essential effect on intestinal probiotics [104]. Wu et al. prepared Pacific abalone sulfate polysaccharide (AGSP) and its desulfurization product (D-AGSP) and analyzed the effect of the sulfate group. The results showed that the abundance of probiotic bacteria in the AGSP group was significantly higher than that in the D-AGSP group, and the content of butyric acid in the feces of mice in the D-AGSP group was lower than that in the AGSP group [118]. Sulfate groups partially determine the hypoglycemic activity of polysaccharides through bacteria-mediated pathways, but the specific mechanism needs further study.

#### 4.2.2. Substitution Position of the Sulfate Group

SC-FUC is a linear polysaccharide consisting of regular disaccharide, trisaccharide or tetrasaccharide repeated units with clear glycoside bonds and unique sulfation patterns [119]. The sulfate group pattern (2-*O*-, 4-*O*- or 2,4-*O*-sulfate group substitution) and the position (in ortho fucose or meso fucose) are important factors affecting its activity [82,119]. Hu et al. studied the effects of sulfation position on improving insulin resistance from five SC-FUC from different sources. It is inferred that meso-fucose is better at alleviating IR, and 4-*O*-sulphate substitution in SC-FUC is more beneficial than 2-*O*-sulphate substitution is at alleviating IR [85]. Studies have also shown that the 4-*O*-sulfated structure is vital in treating metabolic syndrome functional groups [77,120]. Li et al. suggested that the significant effect of Fuc-*Pg* on reducing obesity and improving blood lipids is related to the high amount of 4-*O*- sulphate substitution [120,121].

The bioactivity of SC-FCS is also closely related to the sulfation mode of its backbone. Most of the sulfate groups of FCS are attached to C-4 and/or C-6 of GalNAc residues and C-2 of GlcA residues [122]. The common combinations of sulfation patterns of its backbone include GlcA-GalNAcp4*S* (CS-A unit), GlcA-GalNAcp6*S* (CS-C unit) and GlcA-GalNAcp4*S*6*S* (CS-E unit). In addition, GlcA-GalNAc (CS-O unit) and GlcAp2*S*-GalNAcp6*S* (CS-D unit) are also present, but in smaller proportions. The sulfation of the fucose branch occurs mostly at the C-4 position, and its sulfation patterns include Fucp2*S*4*S*, Fucp3*S*4*S*, and Fucp4*S*, of which Fucp2*S*4*S* is important for SC-FCS activity [94,123]. The sulfate content of most FCS accounted for about 30–40%, and the differences in sulfate content of different FCSs were small; the hypoglycemic effect and mechanism of SC-FCS are different due to different sulfate acidification modes [74,92,99,123]. fCS-Ib from *I. badionotus* has a backbone that consists of a repeating disaccharide unit [4GlcAβ1-3GalNAc(4,6*S*)β1] with sulfate groups attached to the C-4 and C-6 positions of GalNAc residues and the presence of a single Fucp2*S*4*S* fucose branch attached to the C-3 position of GlcA [124]. fCS-*Ib* not only improves the symptoms of hyperglycemia in HFSD mice, but it also effectively regulates the dysbiosis of intestinal microflora [79]. The FCS structure obtained from *C. frondosa* was identified as →3)-β-D-GalNAc4*S*6*S*-(1→4)-β-D-GlcA3*S*-(1→and→3)β-D-GalNAc4*S*-(1→4)-β-D-GlcA3*S*-(1→, with -L-Fuc*p*3*S*4*S* and-L-Fuc*p*2*S*4*S* branched on Glc*p*A residue *O*-3 and -L-Fuc*p* branched on Gal*p*NAc residue *O*-6 [125]. Zhu et al. evaluated the hypoglycemic effect of CFPF, an FCS fraction extracted from *C. frondosa*. The results showed that CFPF could reduce fasting glucose levels, improve glucose tolerance, improve IR and promote hepatic glycogen accumulation by activating the IRS/PI3K/Akt signaling pathway and regulating GSK-3β gene expression [82]. AHG extracted from *A. japonicus* can alleviate insulin-resistant hepatocyte gluconeogenesis, whose backbone consists of →4)-GlcUAβ-(1→3)-GalNAcβ-(1→ repeat units, and it was sulfated at the *O*-4 and/or *O*-6 positions. And the branches of sulfated fucose occur at *O*-3 of GlcUA or *O*-4/6 GalNAc [78,126]. However, the analysis of the complex structure of some FCS is not extensive enough, and the specific mechanism of sulfation mode on the hypoglycemic activity of polysaccharides is unclear. Therefore, the effect of sulfation mode on the activity of sulfated polysaccharides is an important direction to study in the future.

Many results have showed that the sulfate groups of FU are mainly located at C-2 and C-4 positions, but there are fewer at C-3 positions [95,127,128]. However, there are a few studies on the effect of FU sulfate group location on hypoglycemic activity. Therefore, the relationship between sulfate group content and location and marine fucosyl-polysaccharides activity should be considered comprehensively in subsequent studies.

### 4.3. Monosaccharide Composition

Some researchers suggest that monosaccharide composition also affects the hypoglycemic activity of marine fucosyl-polysaccharides. FU composition usually contains a high proportion of fucose and galactose residues, as well as varying proportions of other neutral and acidic monosaccharides, including mannose, glucose, xylose, glucuronide and galacturonic acid [96,127,128]. SFP-1 (8.47 kDa) and SFP-2 (84.99 kDa) from *S. fusiforme* have the same monosaccharide composition, but in significantly different proportions. SFP-1 comprises 80.04% glucose, 5.71% mannose, 4.03% galactose and 4.93% glucuronic acid. SFP-2 comprises 41.22% fucose, 19.27% galactose, 16.79% mannose and 13.40% glucuronic acid. Both SFP-1 and SFP-2 lowered the fasting blood glucose levels in HFD rats. At the same time, SFP-2 had more prominent hypoglycemic effects by regulating genes related to glucose uptake and utilization and hepatic glucose production. The high content of fucose, galactose and glucuronic acid in SFP-2 may be the reason for the difference in hypoglycemic activity, but the specific SAR needs to be further investigated [50,51]. Shan et al. evaluated the inhibitory effect of FU from different sources on α-glucosidase. For FU with the same type of glycosidic bond, the higher proportion of fucose there is in its monosaccharide composition, the better the inhibitory effect will be [69]. Other experiments have shown that marine fucosyl-polysaccharides with a high ratio of fucose to galactose and other monosaccharides can ameliorate diabetes by modulating the gut microbiota. HLP extracted from *H. leucospilota,* the contents of fucose and galactose were 35.72% and 8.43%, respectively, improved diabetes by regulating intestinal microflora in multiple ways [81,127]. SFF obtained from *S. fusiforme* contains 55.67% fucose and 20.83% galactose, which alters the abundance of probiotics in the gut and reduces pathogenic bacteria, improving diabetes by reducing IR [59]. It is difficult to observe clear regularity because the polymers of marine fucosyl-polysaccharides are heterogeneous, and their proportions vary depending on the extraction process employed [129]. Therefore, it is hard to accurately evaluate the structure–activity relationship of the monosaccharide composition.

### 4.4. Conformation

Some studies have shown that the conformation of polysaccharides also affects their hypoglycemic activity. The conformation of polysaccharide refers to the shape and size of polysaccharide molecules in a solution, including monosaccharide conformation, flexibility and spatial structure. According to the different conformations of polysaccharides in a solution, polysaccharides can be divided into random coils, single helices, double helixes, triple helices, worm-like shapes, rod-like shapes and aggregates [130]. Among them, trihelix polysaccharide is the focus of research, which may confer a higher biological activity level on polysaccharide [131,132,133]. In a study by Cao et al., SPP-1 (1518.6 kDa) and SPP-2 (50.6 kDa) had the same monosaccharide composition and similar ratios, but SPP-1 had better α-glucosidase inhibition and the ability to promote glucose consumption in HepG2 cells. The results suggest that this may be related to the triple helix structure of SPP-1 [44]. However, the current research on the conformation and hypoglycemic activity of polysaccharides is very limited; we could obtain some insights by referring to other kinds of polysaccharides. Chen et al. enzymatically hydrolyzed ALG, and the products (PDA1-4) were obtained with increased enzyme concentrations with increasing degradation, while changing the conformation and viscosity of ALG in the solution. The results indicate that PDA4 has better glucose adsorption and diffusion retardation capacities than ALG and other PDAs do, which is related to the increase in the M ratio in PDA4 to form flexible chains. This conformation increases the internal porosity of PDA4, exposing more surface area for contact with glucose, resulting in more significant glucose adsorption, while effectively inhibiting glucose diffusion, and thus, having potential hypoglycemic effects [61]. Due to the effect of conformation on polysaccharides hypoglycemic activity, this structure–activity relationship of marine fucosyl-polysaccharides should be paid more attention in the future.

### 4.5. Type of Glycosidic Bond

Shan et al. showed that FU of type I had no inhibitory effect on α-glucosidase, while FU of type II had a significant inhibitory effect on α-glucosidase [69]. However, the correlation between the (1→3) (1→4)-linked FU inhibition on α-glucosidase and its structural properties remains uncertain. Kim et al. conducted the in vitro hypoglycemic evaluation of FU obtained in different seasons and periods and obtained similar results. FU obtained from *Ascophyllum nodosum* was mainly linked to α-(1→3), and had a small proportion of (1→4)- or (1→3)- and (1→4)- linked repeats. The IC_50_ value of FU from *Ascophyllum Nodosum* was 0.013–0.047 mg/mL. Additionally, α-amylase also has an apparent inhibitory effect. However, the FU extracted from *Fucus vesiculosus* was only composed of (1→3) units, and the inhibitory rate of this FU to α-glucosidase was generally lower than 50% in different seasons and periods, and it had no inhibitory effect on α-amylase [134]. At present, the structures of many marine fucosyl-polysaccharides are not deeply understood, and their glycosidic bond types are not clear enough for further comparative analysis.

Therefore, the hypoglycemic effect of polysaccharides should not be evaluated by considering a single SAR and integrating it with other structural information. However, the current research on the molecular structure of marine fucosyl-polysaccharides is focused on the primary structure level, and studies of the SAR are limited. The relationship between marine fucosyl-polysaccharides’ advanced structure, spatial conformation and hypoglycemic activity should be further investigated.

## 5. Effect of Marine Fucosyl-Polysaccharides on Diabetic Vascular Complications

Persistent hyperglycemia triggers multiple metabolic signaling pathways, leading to inflammation, cytokine secretion and cell death, resulting in hyperglycemic complications [19]. Complications from DM are generally classified as acute and chronic. Acute complications include diabetic ketoacidosis, a hypertonic hyperglycemic coma and a hypoglycemic coma, while chronic complications mainly involve vasculopathy, including macroangiopathy and microangiopathy, which is a severe DM complication. Hyperglycemia and vascular complications are a two-way vicious cycle process. Not only does hyperglycemia cause vascular dysfunction, but vascular lesions also precede and contribute to hyperglycemia in T2DM by impairing insulin-mediated glucose handling and possibly insulin secretion [19]. In addition, the complications of DM do not exist independently. Still, the molecular mechanism of these complications and their interaction mechanism need further study; especially, vascular complications should be paid more attention. Some marine fucosyl-polysaccharides can not only have a direct hypoglycemic effect, but also an effect on hyperglycemic complications. Through clinical trials, FU may play an essential role in regulating various organs of diabetics, thereby inhibiting complications of DM, especially diabetic vascular complications [128].

The primary manifestation of significant vascular disease is cardiovascular disease (CVD) caused by atherosclerosis, which is mainly characterized by changes in vascular homeostasis caused by the dysfunction of endothelial cells and vascular smooth muscle cells [135]. The extensive research on FU in cardiovascular disease has focused on the anticoagulant and antithrombotic activity, anti-inflammatory activity and modulation of vascular cell behaviors [136,137]. Shang et al. found that FU may exhibit potent inhibition of the intrinsic coagulation pathway by targeting the intrinsic coagulation factor Xase [138]. The anti-inflammatory effect of fucoidan is achieved by inhibiting the complement system, binding to selectins and suppressing the activities of several inflammatory enzymes [136]. Recent studies have shown that FU can reduce the production of MMP and reactive oxygen species by suppressing the activation of nuclear factor, κB and c-Jun N-terminal kinase (JNK), thereby reducing the narrowing of the lumen of the damaged vessels [139]. Directing vascular cell behaviors, including promoting endothelial cells (ECs) and angiogenesis, and restoring smooth muscle cells (SMCs) [137]. Vascular ECs are a significant contributor to cardiovascular diseases. FU may protect against ECs by the expression of endothelial dysfunction marker, endothelin-1, and proinflammatory cytokines, TNF-α and interferon-γ (IFNγ) [140]. Abnormal increases in Vascular SMCs can lead to cardiovascular disease, and FU can reduce SMC proliferation and migration [138,141]. Fucoidan for cardiovascular applications, mechanisms and future perspectives was reviewed by Yao et al and Zaporozhets et al. [136,137].

The common diabetic microvascular complications include diabetic nephropathy (DN) and diabetic retinopathy (DR) [142]. Previous studies have shown that FU can improve diabetic nephropathy in various ways. LMWF can reduce inflammation in DN by maintaining the structural integrity of glomeruli, improving glomerular filtration function, protecting glycosaminoglycans from abnormal degradation and preventing the production of advanced glycosylation products [141]. Recent studies have found that FU regulates diabetic nephropathy via the PKC/NF-κB pathway [143]. Diabetic retinopathy is usually caused by the oxidative damage of retinal cells and pathological changes [144]. Li et al. found that fucoidan protects ARPE-19 cells against high-level glucose-induced oxidative damage via the normalization of ROS generation through the Ca^2+^-dependent ERK signaling pathway, and it also inhibits high-level glucose-induced cell apoptosis [145]. In addition, other marine fucosyl-polysaccharides have ameliorative effects on vascular complications. Rhamnan sulfate extracted from *Monostroma nitidium* has beneficial effects, such as reducing inflammation, binding growth factors and NF-κB, enhancing endothelial barrier function and reducing atherosclerotic plaque formation [146].

## 6. Discussion and Prospects

At present, hypoglycemic drugs have limitations, such as side effects and high prices, and screening natural sources of hypoglycemic ingredients has become a research priority. Therefore, it is necessary to develop natural hypoglycemic products with less toxic side effects. Marine polysaccharides have attracted a lot of attention because of their abundant sources, minimal toxic side effects, high stability and good biocompatibility. It was found that most of the marine polysaccharides with hypoglycemic activity are sulfated polysaccharides containing fucose, mainly from algae and sea cucumbers. Marine fucosyl-polysaccharides can reduce blood glucose by inhibiting digestive enzyme activity, improving insulin resistance, improving β-cells’ function and regulating the gut microbiota. Interestingly, the hypoglycemic activity of marine fucosyl-polysaccharides is closely related to their molecular weight, sulfate group, monosaccharide composition, conformation and the glycosidic bond type of polysaccharides. But, there are still some problems that need to be resolved in the relevant research. The evaluation of the structure–activity relationship of polysaccharides is mostly based on a single structural property. In future studies, different structural properties should be comprehensively considered, and comparisons should also be made between different structures. The limitations of the structure–activity study may be related to purity of polysaccharides, which is difficult to analyze because it is mixed with a variety of substances. Also, the relationship between the advanced structure and spatial conformation of marine fucosyl-polysaccharides and hypoglycemic activity should be further investigated. Moreover, in terms of pragmatic applications, there are many studies on the hypoglycemic effect of marine fucosyl-polysaccharides, but only a few products have been developed. One of the reasons could be that the pharmacology and pharmacokinetics were unclear, and it is worth studying them in the future. Furthermore, the long-term stability of hypoglycemic activity is yet to be investigated. Additionally, compounding marine fucosyl-polysaccharides with existing drugs may reduce the side effects, while maintaining efficacy. This review also suggests that structural modifications, such as metal ions, can also enhance activity and are an option for product development. It is possible that with the advance of biosynthetic pathways and more effective genetic engineering strategies, the industrial-scale production of marine polysaccharide agents could be achieved. In summary, marine fucosyl-polysaccharides have a great advantage in the development of hypoglycemic products and are expected to be sustainable nutritional or functional foods for complementary and alternative treatments for DM.

## Figures and Tables

**Table 1:** Classification and pathogenesis of diabetes mellitus.

Type of Diabetes	Etiology and Pathogenesis
Type I diabetes mellitus (T1DM)	Normal β-cells and antibodies in the body are produced during an immune reaction, causing β-cells to be destroyed, which causes the body to not to be able to secrete the insulin, which, in turn, causes an absolute lack of insulin, leading to abnormal metabolic disease.
Type II diabetes mellitus (T2DM)	(1) A genetic defect in insulin resistance caused by abnormal gene regulation. (2) A genetic defect in beta cells that prevents them from properly secreting insulin. Epigenetic environmental factors, such as obesity, aging, stress, dietary habits, glucose toxicity and lipotoxicity, oxidative stress and endoplasmic reticulum stress, are also closely associated with T2DM.
Gestational diabetes mellitus (GDM)	As a woman gains weight and reduces their physical activity during pregnancy, peripheral insulin resistance develops, and glucose intolerance may occur. This, in turn, undermines pancreatic β-cell function and may contribute to the increased risk of GDM. Behavioral factors, excess weight and inherited genes are associated with insulin resistance, and there is a risk of its further development into T2DM.
Special types of diabetes mellitus	According to their etiology, they are classified into eight major categories: genetic defects of the β-cell, genetic defects in insulin action, diseases of the exocrine pancreas, endocrinopathies, drug- or chemical-induced diabetes, infections, uncommon forms of immune-mediated diabetes and other genetic syndromes sometimes associated with diabetes.

**Table 2:** Structural information and hypoglycemic effect of different sources of marine polysaccharides.

No.	Name	Source		Structural Information				Effect	Reference
				Molecular Weight (kDa)	Monosaccharide Composition	Sulfate Group Content (%)	Structure		
1	LMWF	*Undaria pinnatifida*	Brown algae	1 ± 0.2	-	-	-	Improve glucose homeostasis and insulin resistance due to endoplasmic reticulum stress (in vivo)	[38]
2	-	*Ascophyllum nodosum*	Brown algae	637	Fucose/Galactose/Xylose/ Mannose/Glucuronic acid = 31.1/4.1/6.4/2.9/2.8	20.6	Fucosyl residue/Galactosyl residue/Xyloxyl residue = 82.9/1.2/13.9	The inhibition rate of α-amylase was 83.2% (in vitro)	[39]
3	STP-1	*Sargassum thunbergii*	Brown algae	190.4	Arabinose/Galactose/Gluco-se/Xylose/Mannose/Galact-uronic acid/Glucuronic acid = 1.94/30.7/4.54/23.2/17.6/8.11/13.9	15.2	-	The inhibitory rate of α-glucosidase was 75.0%, which increased the glucose consumption of HepG2 cells (in vitro)	[40]
4	-	*Kappaphycus alvarezii*	Red algae	-	-	-	→4)-4-*O*-sulfonato- (2-*O*-methyl)-β-D-galactopyranosyl-(1→4)-3,6-anhydro-(2-*O*-methyl)-α-D-galactopyranan	The IC_50_ of α-amylase and α-glucosidase were 0.15 mg/mL and 0.09 mg/mL, respectively (in vitro)	[41]
5	-	*Gracilaria opuntia*	Red algae	-	-	-	→3)-4-*O*-sulfonato-(6-*O*-acetyl)-β-D-galactopyranosyl-(1→4)-3,6- anhydro-(2-*O*-sulfonato)-α-D-galactopyranosyl-(1→3)-4-*O*-sulfonato-(6-*O*-acetyl)-β-D-xylosyl-(1→3)-4-*O*-sulfonato-(6-*O*-acetyl)-β-D-galactopyranosyl-(1→4)-3,6-anhydro-(2-*O*-sulfonato)-α-D-galactopyranan	The IC_50_ of α-amylase and α-glucosidase were 0.04 mg/mL and 0.09 mg/mL, respectively (in vitro)	[41]
6	GLP	*Gracilaria lemaneiformis*	Red algae	21.2	Rhamnose/Arabinose/ Xylose/Mannose/Glucose/Galactose/Uronic acids	19.64	-	The IC_50_ of α-amylase is 3.94 μg/mL, which can regulate glucose and lipid metabolism, repair β-cells, protect liver and kidney function, and promote the activity of endogenous antioxidant enzymes (in vitro and in vivo)	[42]
7	GLPs-SeNPs	*G. lemaneiformis*	Red algae	382.3	Fucose/Galactose/Glucose/Xylose/Ribose/Glucuronic acid			The IC_50_ of the α-amylase was 1.550 mg/mL and the IC_50_ of α-glucosidase was 2.139 mg/mL (in vitro)	[43]
8	SPP-1	*Sargassum pallidum*	Brown algae	1518.6	Fucose/Arabinos/Galactose/Mannose/Glucose/Xylose/Glucuronic acid/Galacturonic acid = 4.97/1.00/9.75/6.44/1.71/1.82/6.07/2.20	2.61	-	α-amylase and -glucosidase inhibitory activities, and remarkably improve glucose consumption in insulin resistance (IR) model cells (in vitro)	[44]
9	PSP-2	*S. pallidum*	Brown algae	144.8	Fucose/Arabinose/Galactos-e/Glucose/Xylose/Mannose/Galacturonic acid/Glucuronic acid = 21.6/2.5/22.4/2.2/18.8/1.2/7.7/23.6	4.71	→1)-β-D-Xyl*p*-(3→, →1,3)-β-L-Fuc*p*-(4→, →1)-α-D-Gal*p*-(6→, and →1)-α-D-Glc*p*NAc-(2→, and the side chains were composed of →1,3,6)-α-D-Gal*p*-(2→, →3)-β-L-Fuc*p*-(1,4→, β-D-Gal*p*NAc-(1→, and α-D-Man*p*-(1→	Improve insulin resistance HepG2 cell glucose consumption, promote glycogen synthesis and improve insulin resistance (in vitro)	[45]
10	S-SPP_1-8_	*S. pallidum*	Brown algae	1734	Fucose/Arabinose/Galactos-e/Glucose/Xylose/Mannose/Galacturonic acid/Glucuronic acid = 51.72/5.50/13.74/0.81/1.32/6.60/10.31/9.99	13.36	-	The inhibition rate of α-glucosidase was 98.4%, improve insulin resistance (in vitro)	[46]
11	PSP-1	*S. pallidum*	Brown algae	1036	Fucose/Arabinose/Galactos-e/Glucose/Xylose/Mannose/Galacturonic acid/Glucuronic acid = 18.45/2.15/19.06/1.89/16.07/1.00/5.74/20.09	10.69	-	It has certain inhibitory effect onα-amylase and -glucosidase, improve insulin resistance HepG2 cell glucose consumption (in vitro)	[47]
12	Se-SPP	*S. pallidum*	Brown algae	3610, 1630	Fucose/Arabinose/Galactos-e/Glucose/Xylose/Mannose/Galacturonic acid/Glucuronic acid = 32.13/1.15/19.39/21.0/4.42/11.61/1.99/8.31	-	-	The IC_50_ of α-glucosidase were 0.896 mg/mL (in vitro)	[48]
13	SCO	*Sargassum confusum*	Brown algae	-	Fucose/Arabinose/Xylose/Glucose	-	SCO was a sulfated oligosaccharide containing one Gal unit and one anGal unit, sulfated galactose, sulfated anhydrogalactose and methyl sulfated galactoside units.	Improve insulin resistance and regulate intestinal microflora (in vivo)	[49]
14	SFP-2	*Sargassum fusiforme*	Brown algae	84.99, 14.33	Fucose/Mannose/Rhamnose/Glucose/Galactose/Glucuronic acid = 41.22/16.79/2.65/6.67/19.27/13.40	3.24	-	Improve hyperinsulinemia and insulin resistance, regulate intestinal microflora (in vivo)	[50,51]
15	SFF	*S. fusiforme*	Brown algae	-	Mannose/Rhamnose/ Glucose/Glucuronic acid/Galacturonic acid/Galactose/Xylose/Fucose = 10.89/3.29/4.32/4.53/14.02/18.33/3.57/41.05	17.36	-	Improve insulin resistance and sensitivity, reduce fasting blood glucose and IR index along with improve glucose tolerance, increase the abundance and diversity of gut microbiota, improve intestinal integrity and inflammation (in vivo)	[52]
16	-	*Ecklonia maxima*	Brown algae	10	Fructose/Fucose/Galactose/Glucose/Mannose/Xylose = 12.78/4.45/1.44/1.09/4.30/0.79	6.01	-	The range of α-glucosidase IC_50_ is 0.27–0.31 mg/mL (in vitro)	[53]
17	PD-1	*Porphyra* spp.	Red algae	2.59	Galactose/Glucose = 98.6/1.4	-	-	The IC_50_ of α-amylase was 12.72 mg/mL, and the inhibitory activity of α-amylase was about 98.78% (in vitro)	[54]
18	EPs	*Porphyridium cruentum*	Red algae	-	-	-	-	The inhibition rate of α-glucosidase was 71.57%, increase the number of pancreatic beta cells (in vitro and in vivo)	[55]
19	Up4	*U. pinnatifida*	Brown algae	41.4	Mannose/Rhamnose/Galact-ose/Fucose/Glucuronic acid	8.74	Both α-configuration and β-configuration exist	The IC_50_ of α-glycosidase was 50.5 µg/mL, improve insulin resistance HepG2 cell glucose consumption, Lower fasting blood glucose, improve glucose metabolism disorder, improve insulin sensitivity, increase liver glycogen synthesis (in vitro and in vivo)	[56]
20	MAP	*Macrocystis pyrifera*	Brown algae	472.2, 137.6, 26.8	Fucose/Mannose/Rhamnose/Glucose/Galactose/Xylose/Glucuronic acid = 27.75/26.43/2.14/1.11/6.54/18.77/17.26	7.18	-	It has a positive effect on the control of LDL-C level in diabetic rats, regulation of glucose metabolism and intestinal microflora (in vivo)	[57,58]
21	SFF	*S. fusiforme*	Brown algae	205.8	Fucose/Mannose/Rhamnose/Glucose/Galactose/Xylose = 55.67/4.45/3.34/5.44/20.83/3.70	14.55	-	Reduce fasting blood glucose, inhibit oxidative stress, regulate intestinal microflora (in vivo)	[59]
22	UPP	*U. pinnatifida*	Brown algae	185.5	Fucose/Galactose/ Glucuronic acid/Mannose/Glucose/ Rhamnose/Galacturonic acid = 33.46/29.49/19.17/12.30/2.06%/1.94%/1.58%	-	-	Reduce the level of fasting blood glucose, relieve insulin resistance and regulate the abundance of intestinal microflora (in vivo)	[60]
23	PDA4	*-*	Brown algae	40	-	-	M/G = 1.8, it appears as random coil and compact spherical coil in solution	It has the ability of glucose adsorption and diffusion inhibition (in vitro)	[61]
24	SFP-7-40	*S. fusiforme*	Brown algae	41.27	Fucose/Mannose/Rhamnose/Xylose/Glucuronic acid = 2.5/33.20/2.5/18.02/43.78	32.81	-	The IC_50_ of α-glycosidase was 0.304 mg/mL (in vitro)	[62]
25	CDDP	*Dictyopteris divaricata*	Brown algae	63.06	Mannose/Ribose/ Rhamnose/Glucuronic acid/Glucose/Galactose/ Xylose/Arabinose/Fucose = 15.02/9.90/1.28/17.54/1.86/17.19/4.54/0.55/32.13	-	-	Improves fasting blood glucose and insulin abnormalities, modulates the gut microbiota and maintains the integrity of the gut barrier (in vivo)	[63]
26	ULP-1	*Ulva lactuca*	Green algae	62.12	Mannose/Rhamnose/ Glucuronic acid/Glucose/Galactose/Arabinose/Xylose = 0.22/22.88/9.41/0.44/0.50/3.44/0.60	8.99	(ULP-1) comprised β-D-Xyl*p*-(1→3)-β-D-Ara*p*-(1→6)-β-D-Gal*p*-(1→6)-β-D-Glc*p* linked to [→α-L-Rha*p*-(1→4)-β-D-Glc*p*A→]_n_ and α-D-Man*p*-(1→4)-α-L-Rha*p*(2SO_3_-)-(1→2)-α-L-Rha*p*(4SO_3_)-(1→2)-α-L-Ara*p*-(1→2)-α-L-Rha*p*-(1→as its side chains at β-D-Glc*p*	Improve glucose tolerance and regulate intestinal microflora (in vivo)	[64]
27	LMWAs-H	*A. Nodosum*	Brown algae	33.48	-	-	→4)-α-L-Fuc*p*-(1→4)-α-L-Fuc*p*-(1→3)-β-D-Xyl*p*-(1→3)-α-L-Fuc*p*4S(1→ as main chain, and T-α-D-Glc*p*-(1→ and →3)-β-D-Man*p*Ared residues were attached to the ends of main chain as non-reducing- and reducing-end residues, respectively, the 4-deoxy-L-erythro-hex-4-enuronosyluronate linked the *O*-4 position of →3,4)-β-D-Man*p*Ared residue as side branches	The IC_50_ values of α-amylase and α-glucosidase were 1150 ± 10 μg/mL and 560 ± 10 μg/mL, respectively (in vitro)	[65]
28	LF2	*Laminaria japonica*	Brown algae	7.2	Fucose/Mannose/Rhamnose/Xylose/Galactosamine/Gl-ucose/Galactose/Arabinose	29.3	The backbone was (1→3)-linked α-L-fucopyranose residues and a few (1→4)-α-L-fucopyranose linkages. The branch points were at C-4 of 3-linked α-L-fucopyranose residues by β-D-galactopyranose unites or at C-2 of 3-linked α-L-fucopyranose residues by non-reducing terminal fucose unites	Reduce fasting blood glucose, improve insulin secretion and metabolic syndrome, regulation of intestinal microflora (in vivo)	[66]
29	PSP3	*Spirulina platensis*	Blue-green algae	10–30	-	12.01	-	The IC_50_ of α-glycosidase was 0.85 mg/mL, improve oral glucose tolerance and insulin resistance (in vitro and in vivo)	[67]
30	-	*U. pinnatifida*	Brown algae	-	Fucose/Galactose/Glucose/Glucuronic acid	22.83	The backbone was a repeating structure of alternatively linked α-(1–3) and α-(1–4) fucose and galactose units, with a high degree of sulfation	Inhibit α-glycosidase and α-amylase activities (in vitro)	[68]
31	FvF	*Fucus vesiculosus*	Brown algae	-	Fucose/Galactose/ Xylose/Glucose	-	1→3/1→4 Linkage mode	The IC_50_ of α-glycosidase was 67.9 μg/mL (in vitro)	[69]
32	Am-FUC	*Acaudina molpadioides*	Sea cucumber	1614.1	Fucose	26.3	[→3-α-L-Fuc*p*-1→3-α-L-Fuc*p*2,4(OS_3_^−^)-1→3-α-L-Fuc*p*-1→3-α-L-Fuc*p*2(OS_3_^−^)-1]_n_	Improve hyperglycemia and insulin resistance, regulates intestinal microflora (in vivo)	[70,71]
33	*Cf*-CHS	*Cucumaria frondosa*	Sea cucumber	14.76	Glucuronic acid/Galactosamine/Fucose	30.07	The backbone was CHS E, (4-β-D-GlcA-1→3-β-D-GalNAc)_n_	Improve insulin sensitivity and insulin resistance, repair pancreatic islets apoptosis (in vivo)	[72,73,74]
34	CHS	*A. molpadioides*	Sea cucumber	21.53	Glucuronic acid/Galactosamine/Fucose	27.81	-	Increased insulin signaling pathway, improve glucose metabolism (in vivo)	[75]
35	*Ib*-FUC	*Isostichopus badionotus*	Sea cucumber	450	Fucose	32.9	[→3Fuc(2*S*,4*S*)α1→3Fuc(2*S*)α1→ 3Fuc(2*S*)α1→3Fucα1→]_n_	Improve insulin resistance and inhibite inflammatory response (in vivo)	[76]
36	fuc-*Pg*	*Pearsonothuria graeffei*	Sea cucumber	-	Fucose	-	-	Regulates metabolism and intestinal microflora (in vivo)	[77]
37	AHG	*Apostichopus japonicus*	Sea cucumber	98.07	Glucuronic acid/N-acetylgalactosamine/Fucose	33.2	The backbone structure of→4)GlcUAβ(1→3)GalNAcβ(1→, with *O*-4 and/or *O*-6 positions of sulfation. The sulfated fucose branches occurred at the *O*-3 position of the D-GlcUA moiety or the *O*-4/6 position of D-GalNAc	Improve insulin resistance (in vivo)	[78]
38	fCS-*Ib*	*I. badionotus*	Sea cucumber	10.9	Glucuronic acid/N-acetylgalactosamine/Fucose	-	-	Reduce fasting blood glucose, reduce inflammation, regulate intestinal microflora (in vivo)	[79]
39	HLP	*Holothuria leucospilota*	Sea cucumber	52.80	Rhamnose/Fucose/Glucuro-nic acids/Galactose/Glucose/Xylose = 39.08/35.72/10.72/8.43/4.23/1.83	-	-	Lower fasting blood glucose, regulate metabolic pathways and intestinal microflora (in vivo)	[80,81]
40	TAPF	*Thelenota ananas*	Sea cucumber	1284	Fucose	13.15	[→3-α-L-Fuc*p*-1→3-α-L-Fuc*p*-1→3α-L-Fuc*p*2, 4 (OSO_3_^−^)-1→3-α-L-Fuc*p*2 (OSO_3_^−^)-1→]_n_	Lower fasting blood glucose, improve glucose tolerance, promote insulin secretion or enhance insulin sensitivity, improve insulin resistance and promote liver glycogen accumulation (in vivo)	[82]
41	CFPF	*C. frondosa*	Sea cucumber	30	Glucuronic acid/N-acetylgalactosamine/Fucose	8.21	→3)-β-D-GalNAc4*S*6*S*-(1→4)-β-D-GlcA3*S*-(1→ and →3)-β-D-GalNAc4*S*-(1→4) -β-D-GlcA3*S*-(1→		
42	FCS*sj*	*Stichopus japonicas*	Sea cucumber	60.99	Glucuronic acid/Galactosamine/Fucose	3.71	-	Improve glucose uptake and glucose consumption of HepG2 cells and promote glycogen synthesis of HepG2 cells (in vitro)	[83]
43	NP*sj*	*Stichopus japonicus*	Sea cucumber	301.75	Glucose	-	(1→4)-α-D-glucoses with β-D-glucose (1→) branches substituted at *O*-6 every 7–9 of 1, 4 linked glucoses	Improve glucose uptake and glucose consumption of HepG2 cells and 3 T3-L1 cells model, improve insulin resistance (in vitro)	[84]
32	*Am*-FUC	*A. molpadioides*	Sea cucumber	1614.1	Fucose	26.3	[→3-α-L-Fuc*p*-1→3-α-L-Fuc*p*2,4(OS_3_^−^)-1→3-α-L-Fuc*p*-1→3-α-L-Fuc*p*2(OS_3_^−^)-1]_n_	Lower fasting blood glucose, eliminate insulin resistance (in vivo)	[85]
44	*Ib*-FUC	*I. badionotus*	Sea cucumber	435.3	Fucose	32.9	[→3-α-L-Fuc*p*2(OS_3_^−^)-1→3-α-L-Fuc*p*2,4(OS_3_^−^)-1→3-α-L-Fuc*p*2(OS_3_^−^)-1→3-α-L-Fuc*p*-1]_n_		
45	*Ta*-FUC	*T. ananas*	Sea cucumber	1380.0	Fucose	28.2	[→3-α-L-Fuc*p*2(OS_3_^−^)-1→3-α-L-Fuc*p*2,4(OS_3_^−^)-1→3-α-L-Fuc*p*-1→3-α-L-Fuc*p*-1]_n_		
46	*Ht*-FUC	*Holothuria tubulosa*	Sea cucumber	1567.6	Fucose	27.4	[→3-α-L-Fuc*p*-1→3-α-L-Fuc*p*2,4(OS_3_^−^)-1→3-α-L-Fuc*p*2(OS_3_^−^)-1→3-α-L-Fuc*p*2(OS_3_^−^)-1]_n_		
47	*Pg*-FUC	*P. graeffei*	Sea cucumber	310.5	Fucose	28.2	[→3-α-L-Fuc*p*-1→3-α-L-Fuc*p*2,4(OS_3_^−^)-1→3-α-L-Fuc*p*-1→3-α-L-Fuc*p*4(OS_3_^−^)-1]_n_		

## Data Availability

No new data were created or analyzed in this study. Data sharing is not applicable to this article.
